# The Sustainable Use of Halophytes in Salt-Affected Land: State-of-the-Art and Next Steps in a Saltier World

**DOI:** 10.3390/plants13162322

**Published:** 2024-08-20

**Authors:** Nadia Bazihizina, Jutta Papenbrock, Henrik Aronsson, Karim Ben Hamed, Özkan Elmaz, Zenepe Dafku, Luísa Custódio, Maria João Rodrigues, Giulia Atzori, Katarzyna Negacz

**Affiliations:** 1 Department of Biology, Università degli Studi di Firenze, Via Micheli 1, 50121 Florence, Italy; 2 Institute of Botany, Leibniz University Hannover, Herrenhäuser str. 2, D-30419 Hannover, Germany; jutta.papenbrock@botanik.uni-hannover.de; 3 Department of Biological and Environmental Sciences, University of Gothenburg, P.O. Box 461, 405 30 Gothenburg, Sweden; henrik.aronsson@bioenv.gu.se; 4 Laboratoire des Plantes Extrêmophiles, Centre de Biotechnologie de Borj Cedria, BP 901, Hammam Lif 2050, Tunisia; karim.benhamed@cbbc.rnrt.tn; 5 Department of Animal Science, Faculty of Veterinary Medicine, Mehmet Akif Ersoy University, Burdur 15030, Türkiye; elmaz@mehmetakif.edu.tr; 6 Faculty of Economy and Agribusiness, Agricultural University of Tirana, 1029 Tirana, Albania; zdafku@ubt.edu.al; 7 Centre of Marine Sciences (CCMAR/CIMAR LA), Campus of Gambelas, University of Algarve, 8005-139 Faro, Portugal; lcustodio@ualg.pt (L.C.); mjrodrigues@ualg.pt (M.J.R.); 8 Institute for Sustainable Plant Protection, Consiglio Nazionale delle Ricerche, Via Madonna del Piano, 10, 50019 Sesto Fiorentino, Italy; giulia.atzori@ipsp.cnr.it; 9 Institute for Environmental Studies, Vrije Universiteit Amsterdam, De Boelelaan 1111, 1081 HV Amsterdam, The Netherlands; k.e.negacz@vu.nl

**Keywords:** climate change, cash crop halophytes, phytoremediation, salt-tolerant crop plants, saline agriculture

## Abstract

Salinization is a major cause of soil degradation that affects several million hectares of agricultural land, threatening food security and the sustainability of agricultural systems worldwide. Nevertheless, despite the negative impact of salinity, salt-affected land also provides several important ecosystem services, from providing habitats and nurseries for numerous species to sustainable food production. This opinion paper, written in the framework of the EU COST Action CA22144 SUSTAIN on the sustainable use of salt-affected land, therefore, focuses on the potential of halophytes and saline agriculture to transform and restore key functions of these salt-affected and marginal lands. As the current knowledge on sustainable saline agriculture upscaling is fragmented, we highlight (i) the research gaps in halophyte and salinity research and (ii) the main barriers and potentials of saline agriculture for addressing food security and environmental sustainability in terms of population growth and climate change.

## 1. Agriculture in a Saltier World: Challenges and Opportunities

Salinity is one of the key threats to future food security and sustainability needs. The surface area currently affected by salinization extends for 11 million km^2^, and it is estimated that up to 1.5 million hectares are further lost each year due to this phenomenon [1], while approximately 33% of all global irrigated land has become salt-affected [2]. Estimates indicate that the salinization of irrigated soils alone results in an approximate annual loss of USD 12 billion, and the increasing salinization of soils is among the main factors contributing to soil degradation in Europe, the Middle East, and North Africa [3]. This is the result of unsustainable irrigation practices, climate change, the shortage of suitable agricultural land, and the pressure to use marginal resources due to the competing need for land and water for human use and consumption and non-food crop production. Over the last few decades, climate change has emerged as one of the key drivers in soil and water salinization, especially in coastal regions, where rising sea levels, caused mainly by the melting of ice and the thermal expansion of the ocean due to global warming, affects soil salinization through the intrusion of saline water in aquifers and soils. In parallel, climate-change-induced prolonged drought, heat waves, and warm spells have also led to a further increase in soil salinity, making salinity a new concern for highly productive agricultural regions. For instance, it is estimated that over 50% of European soils have been affected by extreme drought conditions since 2018, with the long, dry heat waves in the European summers of 2022 and 2023 amongst the most severe extreme weather events in recent history [4]. As an emblematic example, the persistent droughts in 2022 led to dramatic saltwater intrusion for tens of kilometers of land in the Po River Delta, an important and highly productive European food production area, which not only affected freshwater provisioning in that area but also irrigation systems and agricultural production in more than 20,000 hectares [5]. Compounding this, most high-yielding crop varieties are salt-sensitive, with the top three primary cereals (wheat, rice, and corn) covering 29% of global agricultural production [6]. Salinity has, thus, been recognized as a serious threat that affects food security and the sustainability of agricultural systems worldwide, with substantial socio-economic repercussions for affected communities.

**Figure 1:**
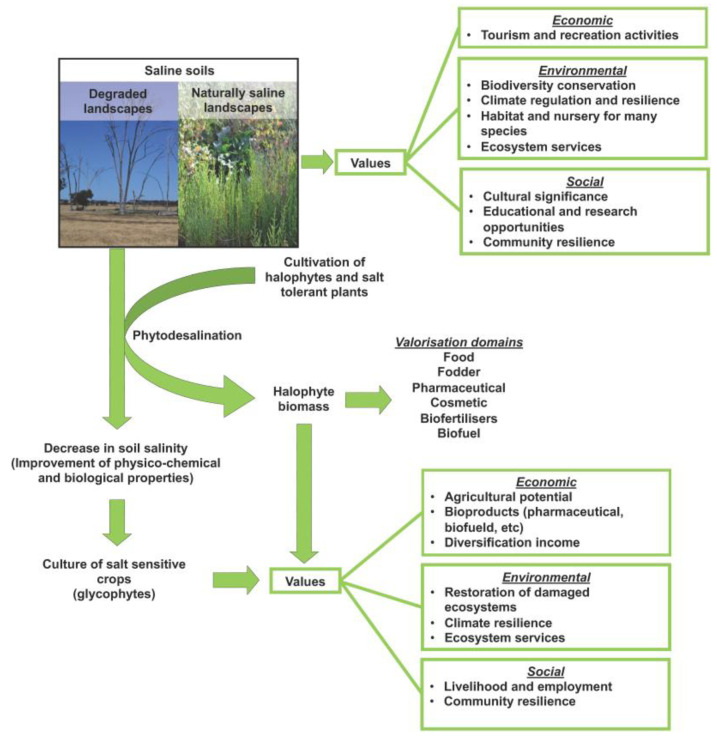
Values and the potential of salt-affected land and saline landscapes. Pictures in the top left show examples of degraded and naturally saline landscapes.

Notwithstanding the negative impact of salinity, salt-affected land provides several important ecosystem services, which are services provided by nature to humans with an economic, environmental, or social impact at different spatial and temporal scales. Indeed, salt-affected land and saline landscapes provide significant values across different aspects, ranging from climate regulation and providing habitats and nurseries for a myriad of species to food production. Furthermore, the sustainable use of saline environments prevents land use changes in other non-degraded ecosystems [7,8]. Indeed, the restoration of salt-affected lands is crucial to making salt-affected ecosystems suitable for use for agricultural purposes. Recently, an FAO publication [9] showed that 1.5 billion people affected by their inability to produce food are living in these marginal environments. Salinization renders around 1.5 million ha of farmland unproductive each year [10] and this is projected to worsen due to ongoing climate change forecasts. In anticipated future circumstances, where salinity is projected to surpass 200 mM NaCl, staple crops are expected to become unproductive [11]. In the future, salt-tolerant halophytes will, therefore, play a vital role in ensuring food security in arid and semi-arid regions. Indeed, halophytes are not only valuable scientific models to improve the performance of traditional crops in salt-affected soils but are also commercially viable alternative crops in saline agriculture, with many species used as food, fodder, or bioenergy crops (see sections below and Figure 1). Furthermore, various alternative agronomic practices worldwide, such as intercropping or rotation, demonstrate the potential of using these plants for restoring saline soils through phytoremediation, specifically halo-phytoremediation [12]. These practices are yielding promising results in terms of both yield and quality for cash crop species. For example, *Arthrocaulon macrostachyum* holds significance for desalination and co-cultivation. When grown in saline soil, it effectively reduces soil salinity by 30% within 30 days [13]. In addition, when co-cultivated with barley, wheat, and tomato, this halophyte reduces the negative effects of salt on the growth of sensitive crops [14]. This could be of great importance in arid and semiarid regions, where insufficient precipitations and drip irrigation systems are unable to reduce the salt content in the rhizosphere. Compared with other techniques, phytoremediation has several unique advantages, including removing salt uniformly, absorbing other pollutants simultaneously, and improving soil quality [15]. Cultivating halophytes not only efficiently restores saline soils but also directly supplies food, fodder, and industrial materials. For example, *Salicornia bigelovii*, a potential oil-seed halophyte for coastal and saline lands, could produce ca. 18 tons ha^−1^ of biomass and 2 tons ha^−1^ of seeds [16]. Introducing sustainable halophyte farming in arid and semi-arid regions will bring new, health-promoting food sources and tackle environmental concerns tied to restoring the biodiversity impacted by human activities and climate change.

The sustainable use of saline environments goes beyond agriculture. On one hand, naturally, saline environments are unique ecosystems that provide refuge to unique fauna and flora, including migratory birds and salt-tolerant plant species. As a result, naturally saline environments are often used for recreational purposes, including walking, hiking, birdwatching, and wild picking, depending on local culture and regulations. In some regions, both food production and tourism are combined in the form of agrotourism (e.g., Terschelling, the Netherlands [17,18]). On the other hand, while there are limited data on the impact of secondary salinization on biodiversity, empirical evidence from stakeholders and researchers working in the field clearly indicates that secondary salinization leads to multitaxon biodiversity collapse [19]. In this context, halophytes offer a valuable tool for managing agriculture to enhance biodiversity and restore ecosystem functions in degraded systems. Although this aspect remains unexplored, understanding how to utilize halophytes to increase plant genetics and functional biodiversity in salt-affected lands, and its associated benefits has the potential to reveal critical new knowledge for the sustainable management of saline landscapes. Therefore, in this opinion paper, initiated by the EU COST Action CA22144 SUSTAIN on the sustainable use of salt-affected lands we focus on the potential of halophytes and saline agriculture to transform and restore the key functions of these salt-affected and marginal lands worldwide, allowing farmers to use these areas to achieve higher yields than possible with conventional management methods or crops [8]. We also discuss open questions and the main barriers in halophyte research and upscaling in agriculture and the implications of addressing these for the development of a saline agriculture capable of addressing food security and environmental sustainability in terms of population growth and climate change.

## 2. Halophytes as Model Species for Understanding Salt Tolerance

Plant growth responds to salinity in two phases: a first and immediate osmotic phase that inhibits water uptake and the growth of young leaves and a slower ionic phase that accelerates the senescence of mature leaves, which can exhibit phytotoxic effects on plants [20]. Salt stress has an impact on plant physiology both in the short- and long-term, affecting plants from a whole-plant perspective down to cellular dynamics due to the combination of osmotic and ionic effects [21,22]. The correlation between yield and root zone salinity has been used as the basis for understanding the whole plant response in saline environments for many crops in most trials investigating salt tolerance [23]. No simple answers derive from these investigations, as a huge variability is present when observing the response of different genotypes to salinity.

Plant species are generally classified into glycophytes and halophytes. Most crop plants fall into the glycophyte category and are sensitive to high-salinity conditions, particularly NaCl. Etymologically, ‘halophytes’ means ‘salt-loving plants’ (hals—salt; phyton—plant; and philein—to love), while ‘glycophytes’ refer to plants that prefer ‘sweet’ substrates (glykos—sweet) [24]. The already mentioned broad spectrum of salt tolerance translates into salt tolerance thresholds ranging from a concentration of 50 mM NaCl up to 500 mM NaCl (circa seawater concentration) [25]. These diverse responses are underpinned by the mechanisms that plants develop to adapt to saline environments [8]. These mechanisms include the synthesis of organic solutes, salt excretion by gland-like structures on shoots (such as salt glands or salt bladders), increased leaf and stem succulence, growth during favorable seasons or in favorable sites, selectivity against Na^+^ and Cl^−^, and sequestering Na^+^ and Cl^−^ ions in vacuoles or other compartments to prevent cytoplasmic toxicity [26,27,28,29].

Despite a wealth of research in the scientific literature investigating plant salt tolerance and related mechanisms, several issues must be resolved before this knowledge can be effectively translated into food production in saline environments. One major challenge is that most salt tolerance trials are conducted under controlled or semi-controlled conditions. When these results are applied to field conditions, differences emerge due to the interaction of multiple variables with the salinity effect. These variables range from the heterogeneity of salinity to other abiotic stresses such as waterlogging, heat, and nutrient deficiencies. This clearly highlights the urgent need for field studies that aim to phenotype selected genotypes under specific salinity and climatic conditions. Addressing this bottleneck is crucial for expanding saline agriculture based on scientific data and stakeholder involvement.

Most studies have focused on the Na^+^ ion due to its well-understood toxicity related to salt stress, but there are still gaps in understanding the roles of other ions, such as Ca^2+^ and K^+^ [30,31]. For example, research has shown that plants coordinate long-distance K^+^ signals between the shoot and root, including efficient K^+^ loading into the xylem for transport to the shoot and the decreased recirculation of K^+^ in the phloem. This coordination is hypothesized to be a signature of improved salt tolerance [31]. Moreover, K^+^ efflux in the cytosol is proposed to act as a molecular switch to turn off energy-consuming processes during stress [32]. Although several energy-related enzymes are linked to activation by K^+^, it remains unclear how they support plants in withstanding salt stress and ensuring continued growth [31]. Additionally, K^+^ is suggested to be linked with Ca^2+^ via K^+^/H^+^ symporters, also known as HAK/KUP transporters [33], as Ca^2+^ levels in the cytosol can regulate or be associated with their activities [34].

Changes in Ca^2+^ levels in the cytosol mediate signals to different target groups, e.g., receptors, scavenger systems, and phytohormones [30]. Although links exist between Ca^2+^ and phytohormones such as ABA and brassinosteroids in salt-stress plants, the exact mechanism behind how brassinosteroids affect Ca^2+^ levels is not yet settled [35]. For instance, uncertainties remain about the exact linkage between Ca^2+^ and ABA related to sensing in the roots and guard cells [36,37]. Calcium signaling is highly complex, relying on interactions with various chemicals and mechanisms [30], and modern gene-editing techniques could help elucidate transduction pathways and genes associated with salt stress.

Finally, silicon (Si) has gained attention for its role in Na^+^ and K^+^ transporters, which is crucial for ion homeostasis in cells during salt stress. One connection is through increased endodermal barriers in the roots, which reduce salt stress in the shoots by limiting the transport and deposition of Na^+^ and K^+^ [38]. Additionally, Si has been shown to enhance the accumulation of Na^+^ in vacuoles by increasing the activity of the NHX transporter in maize, preserving chloroplast integrity, and maintaining photosynthesis [39]. Thus, while Na^+^ requires further study, other less-studied ions also hold the potential for advancing our understanding and mitigation of salt stress.

Progress in engineering salt-tolerant varieties of conventional crops has been limited due to the complex nature of salt tolerance, as it involves multiple genes [40]. In this context, while model organisms (e.g., *Arabidopsis thaliana*) have the advantage of accessible and convenient systems with many resources available, working with salt-loving halophytes has the potential to be exceptionally rewarding because of the promise of new insights into old problems and a whole set of new questions to solve. Indeed, studies on halophytes have played a vital role in the recognition that employing Na^+^ as a ‘cheap osmoticum’ could be an efficient strategy for long-term salt tolerance. Indeed, this ability to use Na^+^ as a cheap osmoticum in shoot tissues has been hypothesized to be energetically less expensive than excluding Na^+^ at the root level, as it minimizes the need for the biosynthesis of organic solutes to achieve full osmotic adjustment and potentially reduces salt accumulation in the rhizosphere in the long-term [41]. Calculations indicate that while the sequestration of Na^+^ (or Cl^−^) ions in root or leaf cells requires ca. 4 to 7 ATP molecules per ion, the ATP requirement for the synthesis of organic compounds is of an order of magnitude that is higher per solute [42]. In accordance with this vision, research, and breeding activities have more recently focused on increasing Na^+^ sequestration within the cell vacuoles of plant tissues, mainly by expressing the tonoplast Na/H antiporter in transgenic plants [43]. Surprisingly, however, in this case, progress is underwhelming, and none of these attempts have delivered salt-tolerant cultivars to farmers’ fields [40,41]. As Na/H exchangers must be energized by vacuolar H pumps, which requires an increased activity of tonoplast H-ATPases and/or H-PPases pumps, this shortcoming is likely to be related to a limited understanding of the costs involved in controlling ion transport across membranes.

Similar to solving the tissue tolerance versus exclusion dilemma, halophytes could also hold the key to defining the ideal root ideotype for saline soils. Indeed, saline soils are inherently heterogeneous, both in time and space, with strong variations in soil water, nutrients, and salinity concentrations [44,45]. Over the last decade, an important effort has been made to understand plant responses to spatially heterogeneous salinities, with the key finding from these studies showing that shoot growth is maintained even when half of the root system is exposed to NaCl concentrations that greatly inhibit growth if applied uniformly. However, key questions remain unanswered, particularly those pertaining to the ideal root ideotype for these inherently heterogeneous saline soils. Are there any specific root traits that might facilitate resource foraging while reducing root metabolic costs? What about salt, water, and nutrient sensing and signaling (short and long-distance signaling) and their interactions with root morphology, anatomy, and physiology in the overall plant performance?

Numerous studies have been conducted to elucidate the salt-sensing and signaling mechanisms responsible for detecting and combating the stress imposed by high salinity [46]. Although an impressive number of proteins have been identified with roles in salt sensing and signaling, there is still room for new discoveries and the clarification of existing findings [46]. It is important to note that several factors could explain diversity in the reported methods of handling salt sensing and signaling. These factors include growth conditions, genetic background within the same species, the plant development stage, the spatial resolution/environment, access to plant material, and the harvested plant part (e.g., root, shoot, or stem). This is compounded by a lack of tools to increase the resolution at the cellular and molecular level to settle the whole chain of events linked to phytohormones and the ion transporter [46]. Furthermore, much of our knowledge of root sensing and signaling has primarily been inferred from studies on glycophytes. Comparative studies indicate that, unlike glycophytes, halophytes rapidly perceive changes in soil salinity and express adaptive responses at the root level [47]. Understanding how halophytes sense and signal salt may reveal specific root traits that facilitate resource foraging in saline soils while reducing root metabolic costs, which is a trait associated with improved ion relations. This knowledge could explain their ability to thrive in hostile and extreme conditions. Additionally, many transporters regulate the flow of ions across membrane boundaries, signaling to hormones (especially ABA) and scavenger pathways such as ROS [48]. This raises the question of whether more hormones and scavenger pathways remain to be investigated in detail.

In conclusion, focusing more on halophytes could enable the identification of genes linked to their tolerance and potentially uncover similar genes in glycophytic crops that could be activated if dormant [41,49,50]. However, it is critical to consider the energetic costs associated with implementing specific salt-tolerance traits from halophytes into salt-sensitive species. Without such consideration, these efforts might ultimately prove futile in enhancing plant salt tolerance [29,51,52]. Understanding the costs of specific traits (e.g., the relative cost of excluding Na^+^ and Cl^−^ in the roots or its use for osmotic adjustment) is, therefore, likely to expedite and facilitate the translation of our knowledge into greater crop yield gains under saline conditions. In this context, new-generation methods, including nanosensors, AI, RNA sequencing (transcriptomics, open-source data), gene editing, and high-density mapping populations (crosses between salt-tolerant and salt-sensitive species) for high-throughput phenotypic platforms, as well as advanced techniques like intense cloning, cell transcriptomics, single-cell sequencing, and 3D electron tomography, are critical. These tools not only predict how genes regulate salt tolerance (e.g., transcription factors that can regulate functional salt genes) but also help avoid the potential yield penalties associated with transferring traits from halophytes to glycophytes.

## 3. The Economic Potential of Halophyte Use: Key Attributes, Challenges, and Main Research Gaps

The use of halophytes as alternative food and non-food crops is a key focus of saline agriculture. Indeed, there is a growing recognition in the scientific, research, and development communities of the limitations of current food production technologies [53]. This perhaps reflects the fact that the crop selection process has been developed without considering the constraints occurring in more marginal and extreme environments. On the contrary, the selection for higher yields under optimal conditions during the green revolution of the late 20th century has dramatically reduced the tolerance of elite crops to abiotic stresses [54]. Fortunately, 450 million years of land plant evolution has generated biological complexity, which has allowed the so-called “extremophiles” to adapt to extreme environments, including halophytes, which has not only resulted in an elevated tolerance to salinity but often also a combined tolerance to other abiotic stresses as their habitats are often prone to flooding, drought, and high temperatures.

Due to their peculiar features, halophytes have been used and consumed by humans for centuries worldwide [55,56]. With ca. 1560 species being classified as halophytes [57], a total of 115 halophyte taxa have been evaluated as food crops and 331 as fodder in various regions and under different climates [58], including *Salicornia*, *Sarcocornia, Sesuvium, Suaeda,* and *Atriplex* spp. to name a few. Important information on most of these halophytes is summarized in the database eHaloph (https://ehaloph.uc.pt/, accessed on 2 August 2024) and can be screened in an interactive way. Recently, the economic uses of all the species listed in the database have been included, which makes it very valuable for non-experts and stakeholders too. Thanks to the capacity of halophytes to thrive in saline lands where non-halophytic species cannot, the cultivation of halophytes is considered an environmentally and economically sustainable alternative for the (re)use of degraded landscapes. This approach reduces the risks of land abandonment and conserves local freshwater resources, particularly in arid regions. Some applications of halophytes can be highly profitable, both for the production of food and fodder crops [59], with clear economic benefits for farming and local communities affected by this issue. For instance, halophytes are being promoted as alternative candidates for bioenergy crops, as both the oil extracted from their seeds and their lignocellulosic biomass can be utilized for biofuel production [55]. Indeed, the potential of creating bioenergy from salt-affected soil has been estimated to have a global technical potential of 56 EJ y^−1^ (ca. 8.5% of the primary energy consumption in 2023 [60]) alongside an economic potential of 21 EJ y^−1^ [61]. Moreover, several halophytic species are rich in dietary components and are, thus, considered fit for integration into the food industry, with a growing market for using halophytes in high-end gourmet cuisine [62]. The most emblematic example of a highly profitable halophyte crop is quinoa (*Chenopodium quinoa*). Quinoa, often hailed as a superfood, has gained significant attention for its exceptional nutritional profile and adaptability to diverse growing conditions. Rich in essential amino acids, vitamins, minerals, and antioxidants, quinoa offers a complete protein source that is particularly beneficial for vegetarian and vegan diets. In 2020, global quinoa production reached approximately 161,000 metric tons, with major contributions from Bolivia and Peru, which together account for over 80% of the world’s supply [9]. The quinoa market size has expanded rapidly in recent years and is projected to increase from USD 101.49 billion in 2023 to USD 112.72 billion in 2024, reflecting a compound annual growth rate (CAGR) of 11.1%. Looking ahead, the quinoa market is expected to continue its rapid expansion, reaching USD 169.54 billion by 2028 with a CAGR of 10.7% [63]. Well-known halophytes are found in the genus *Salicornia*, which has gained consumer interest as a gourmet and functional food due to its rich phytochemical content and associated health benefits. It is consumed worldwide and is either eaten raw in salads or cooked by boiling, steaming, stir-frying, and more, often paired with meats, rice, and pasta. Around 90 bioactive compounds, including saponins, phenols, flavonoids, sterols, and terpenes, contribute to its health-promoting properties such as antioxidant, anti-inflammatory, antidiabetic, anticancer, anti-obesity, cardioprotective, and neuroprotective effects. Commercial production is mainly in the U.S., Mexico, UAE, Germany, and Israel, using seawater irrigation and hydroponics [64]. However, more research is needed to identify the exact species, subspecies, and even ecotypes of several halophytes, especially in the Salicornia genus, and subsequently characterize their ecological and nutritional properties before they can be used as regular crop plants [65,66].

Halophytes are also suitable as fodder crops and for the extraction of natural bioactive substances and health-promoting compounds, with many applications in folk medicine, ethnopharmaceuticals, and ethnoveterinary practices. For instance, in the Near East region, mainly in Egypt, a wide range of halophytes (e.g., *Atriplex* spp., *Kochia* spp., etc.) are considered promising feed resources for small ruminants raised in saline land or arid and semi-arid regions [67]. This is partly because halophytic fodders can serve as drought reserves to fill seasonal feed gaps and can be used as potential sources of nitrogen and major minerals for sheep and goats fed on low-quality diets [59]. Moreover, active ingredients obtained by in vitro sustainable technologies [65] and/or botanical extracts obtained from selected species, such as, for example, *Crithmum maritimum* (sea fennel) and *Armeria maritima* are used in cosmetic formulations sold by several reputed brands [68,69,70,71].

When focusing on halophytes such as crop plants in saline agriculture, it is important to remember that these species have not yet been fully developed or improved through breeding processes or only to a very limited extent. The domestication of wheat began about 10,000 years ago; with increased knowledge and biotechnology, the breeding progress of halophytes can become faster, but this still requires time. Therefore, clear breeding goals must be established first. Achieving higher homogeneity in germination, growth, flowering time, and other parameters is crucial for the successful cultivation of new crop plants. Additionally, halophytes are known to contain several anti-nutrient compounds, such as oxalates, nitrates, phenolics, saponins, and tannins [61], which can have negative health consequences on both food and fodder crops. Thus, breeding for low anti-nutritional compounds should be a research priority. In addition to breeding, sustainable management practices, from species selection to agronomic and post-harvest techniques, need to be optimized or developed, considering the value chain, consumers, and the need to build capacity in local communities. For example, when Salicornia species are cultivated on land influenced by tides, heavy equipment cannot be used, necessitating the development of other technical solutions for land preparation, sowing, fertilizer application, and harvest. Additionally, it has been demonstrated that the presence of anti-nutritional compounds can be mitigated to some extent with specific management practices (e.g., N nutrition that increases in NH_4_^+^ nutrition over NO_3_^−^ nutrition has been linked with oxalate accumulation [61]). Concentrated efforts, such as those in European projects, can accelerate these developments. Decisions must also be made regarding the spectrum of products for food and non-food purposes. Many halophytes offer high nutritional values in different plant organs, such as leaves and fruits; however, a reliable halophytic source of starch, which is a major carbohydrate source for human nutrition, has not yet been identified. This gap needs to be addressed, possibly by modifying starch-producing glycophytes like potatoes or adapting their cultivation conditions, such as through intercropping. Finally, consumer acceptance of new crop plants is crucial, considering factors like taste, preparation methods, health aspects, and market price compared to similar products. Based on the results obtained in customer surveys addressing these points mentioned, marketing strategies have to be developed alongside campaigns highlighting the advantages of halophytes. These have to be specifically adapted on a regional or national level as in some parts of Europe, for example, Northern France, several halophytic species are already well accepted by customers [71], transforming them into unique regional products. Indeed, research in the Netherlands has demonstrated that chefs and restaurant owners can successfully incorporate seasonal halophytes into their menus, leveraging local resources to gain competitive market advantages [18]. Saline recipes are increasingly featured in culinary books like ‘Zilt’, contributing to regional development and raising awareness. These recipes, which highlight halophytes, help create a market niche and stimulate demand for halophyte-based products.

## 4. Halophytes on the European Policy Agenda

European policies related to halophytes encompass various aspects of environmental conservation, agricultural innovation, and sustainable resource management. These policies are often integrated into broader frameworks that address biodiversity, climate change, and sustainable agriculture. There is no single policy framework addressing halophytes specifically.

Within biodiversity conservation, the EU Biodiversity Strategy for 2030 aims to protect and restore biodiversity across EU countries. This strategy includes saline environments such as coastal saline marshes where halophytes thrive. Furthermore, many halophytes grow in areas included in the Natura 2000 Network, protecting unique and threatened habitats. Also, the Water Framework Directive aims to enhance the status of aquatic ecosystems that often include halophytes as well.

The Common Agricultural Policy supports sustainable agriculture practices, which can include halophyte cultivation and soil reclamation using salt-tolerant crops. Halophytes are also supported by the EU Green Deal, which entails sustainable food systems. Also, the EU Adaptation Strategy can be considered supportive for using halophytes as an adaptation strategy to climate change.

Research and innovation are other policy areas in which halophytes are addressed indirectly. In particular, the Horizon Europe and LIFE programs fund projects that address environmental and social challenges, including further research on halophytes. Building the community of research and practice around the theme of the sustainable use of salt-affected land, including halophyte cultivation, is supported by the European mechanism of COST Actions (e.g., https://sustaincostaction.eu/ accessed on 2 August 2024). One major issue in the commercial production of edible halophytes for the European market is missing food-safety regulations, for example, with respect to mycotoxins [66]. This is a major shortcoming of the regular consumption of edible halophytes. Even though there is no single policy addressing halophytes, multiple policy frameworks of the European Union create an enabling environment for the current and future use of these plants.

## 5. Conclusions

While the potential benefits and various uses of halophytes in degraded agricultural land are well-documented, their successful integration into commercial farming faces several challenges. Despite their common use as forage and fodder in regions like North Africa, the Middle East, and Australia, their cultivation is often limited to small-scale or niche productions, especially for high-end restaurants. Expanding beyond these scales and highlighting their potential in salt-affected soils worldwide requires addressing issues related to breeding and agronomic management practices, such as improving yields, reducing anti-nutritional compounds, and enhancing labor efficiency. These challenges are further exacerbated by the lack of comprehensive economic analysis, limited value chain development, and insufficient capacity-building programs. While pilot projects have shown promise, more extensive field experiments and economic evaluations are needed to determine the feasibility of large-scale and global halophyte agriculture. Support and incentives from organizations like the EU or national ministries could encourage the cultivation of halophytes, such as *Salicornia*, involving stakeholders in the design of supportive programs and ensuring consumer acceptance [56].
